# Sympathetic nervous system alterations with HER2+ antagonism: an early marker of cardiac dysfunction with breast cancer treatment?

**DOI:** 10.3332/ecancer.2014.446

**Published:** 2014-07-17

**Authors:** Carrie G Lenneman, Wissam M Abdallah, Holly M Smith, Vandana Abramson, Ingrid A Mayer, Cheri Silverstein, Cheri Silverstein, Julie Means-Powell, Sachin Y Paranjape, Daniel Lenihan, Douglas B Sawyer, Satish R Raj

**Affiliations:** 1 Department of Medicine, Vanderbilt University School of Medicine, Nashville 37232, TN, USA; 2 Department of Medicine, University of Louisville School of Medicine, Louisville, KY 40292, USA; 3 Department of Medicine, University of California Los Angeles School of Medicine, CA 90404, USA; 4 Department of Pharmacology, Vanderbilt University School of Medicine, Nashville TN 37232, USA

**Keywords:** cardio-oncology, catecholamine, heart failure, sympathetic nervous system

## Abstract

**Background:**

HER2 antagonists (anti-HER2; e.g., trastuzumab and lapatinib) are effective in treating an aggressive form of breast cancer (BC), but can cause cardiotoxicity due to the disruption in neuregulin (NRG)/HER2+ ligand receptor signalling. The recent data show that NRG-HER2 receptors located in the medulla oblongata are important regulators of vasomotor tone. Disrupting the NRG-HER2 signalling in mouse medulla results in increased sympathetic nerve output and blood pressure. We hypothesized that anti-HER2 agents would cause increased sympathetic tone with changes in plasma catecholamines and NRG.

**Methods:**

In 15 newly diagnosed HER2+ BC patients receiving anti-HER2 agents, vital signs were measured along with supine plasma epinephrine (EPI), norepinephrine (NE), and NRG at baseline and three months. Serial echocardiography was performed.

**Results:**

With three months of anti-HER2 treatment, NE increased (2.334 ± 1.294 nmol/L vs. 3.262 ± 2.103 nmol/L; *p* = 0.004) and NRG decreased (12.7±15.7 ng/ml vs. 10.9 ± 13.3 ng/ml; *p* = 0.036) with a corresponding increase in systolic blood pressure (110 ± 10 mmHg vs. 120 ± 16 mmHg, *p* = 0.049) and diastolic blood pressure (67 ± 14 vs. 77 ± 10, *p* = 0.009). There was no change, however, in EPI (0.183 ± 0.151 nmol/L vs. 0.159 ± 0.174 nmol/L; *p* = 0.519) or heart rate (73 ± 12 bpm vs. 77 ± 10 bpm, *p* = 0.146). Left ventricular ejection function declined over the follow-up period (baseline 63 ± 6% vs. follow-up 56 ± 5%).

**Conclusions:**

Anti-HER2 treatment results in increased NE, blood pressure, and decreased NRG; this suggests that the inhibition of NRGHER2 signalling leads to increased sympathoneural tone. Larger studies are needed to determine if these observations have prognostic value and may be offset with medical interventions, such as beta-blockers.

**Clinical Trial Registration:**

The study was registered with www.clinicaltrials.gov (NCT00875238).

## Introduction

Trastuzumab (TZB) and lapatanib (LAP) are biological antagonists targeting the HER2/neu receptors (also known as ERBB2 receptor) which have dramatically improved outcomes for HER2+ breast cancer (BC), a variant of the disease which is generally associated with a poor prognosis [1, 2]. Currently, TZB and LAP are used in the treatment of local and advanced HER2+ BC. Both TZB and LAP have been associated with the development of overt heart failure (HF), or asymptomatic cardiac dysfunction, and this is attributed to the disruption in the homeostatic myocardial regulation via neuregulin (NRG)-HER2 signalling. The incidence of cardiotoxicity with HER2 antagonism has been reportedly as high as 34% when used in conjunction with anthracyclines [1, 3–6].

The mechanism of cardiotoxicity associated with HER2 antagonism appears distinct from the oxidative stress-induced apoptosis and necrosis observed with anthracycline-based regimens. The cardiac dysfunction observed with TZB/LAP therapy is thought to occur from disruption in NRG-HER2 signalling. NRG is a critical paracrine factor, expressed by endothelial cells, which activates HER receptors expressed on the myocardial tissue and regulates intracellular signalling required for myocyte growth, survival and repair [7, 8]. NRG is also released in the central nervous system and plays an important role in regulating vasomotor tone and sympathetic output through the rostral ventrolateral medulla (RVLM). In mice, microinjection of NRG into the RVLM results in decreases in arterial blood pressure, heart rate, and sympathetic nerve traffic [9]. In contrast, HER2 inhibitors in mice cause increased arterial pressure and sympathetic nerve activity [9]. Studies demonstrate that HER2 inhibitors in the RVLM cause an increase in blood pressure via modulating the effects of nitric oxide synthase (NOS) [9]. Thus, NRG/HER signalling regulates vascular tone and blood pressure in mice.

We hypothesized HER2-targeted therapy used in treatment of cancer will change vascular tone, as evidenced by changes in vital signs and in circulating plasma catecholamines, as well as plasma NRG. To test this hypothesis, we examined a subset of women with HER2+ BC being followed to assess predictors of cardiotoxicity prospectively during cancer treatment (NCT00875238). We assessed serial vital signs, plasma catecholamines, and NRG, as well as changes in left ventricular ejection function (LVEF) before and during treatment with either TZB or LAP.

## Methods

### Study participants

The study population consisted of a prospective cohort of 15 women with newly diagnosed HER2+ BC undergoing treatment with either TZB or LAP from August 2008 to April 2012 from an urban, academic centre. The patients underwent two study visits followed by yearly phone follow-up interviews and electronic medical record review to ascertain change in cardiac function or the development of symptomatic HF. The current report is from a small sub-study of a larger ongoing clinical trial to explore the biological interaction of HER2+ antagonism on the autonomic nervous system. The sub-study was a small proof of concept study that was planned to guide a larger interventional study. Women were excluded if they had heart failure/cardiac dysfunction (ejection fraction <50%), distant BC metastasis or recurrent BC disease. The Institutional Review Board approved the study (IRB #070927) and it was registered with www.clinicaltrials.gov (NCT00875238). All participants gave their written informed consent prior to any study procedures.

### Cardiac risk factors and blood collection

Baseline data on age, body mass index (BMI), personal history of hypertension, hyperlipidemia, diabetes mellitus, tobacco use, coronary artery disease, and family history were collected at enrollment.

Participants had two study visits at baseline and approximately three months after initiation of anti-HER2 treatment. Vital signs were measured according to protocol by a trained research coordinator, and the blood was collected for fractionated catecholamines and NRG at each visit in the clinical research centre.

### Description of catecholamine and NRG assay

For the catecholamine measurements, the blood was collected in plastic syringes, and immediately transferred to chilled vacuum tubes with sodium heparin (BD, Franklin Lakes, NJ) and placed on ice. Plasma was separated by centrifugation at – 4°C and stored at – 70°C in collection tubes with 6% reduced glutathione (Sigma-Aldrich, Inc, St Louis, MO, USA) until the assay was performed. Concentrations of norepinephrine (NE) and epinephrine (Epi) were measured by batch alumina extraction followed by high-performance liquid chromatography for separation with electrochemical detection and quantification [10]. Plasma norepinephrine and epinephrine are reported in SI units. To convert from nmol/L to the more conventional pg/mL, multiply by 169.18 for norepinephrine (1 nmol/L = 169.18 pg/mL) or by 183.2 for epinephrine (1 nmol/L = 183.2 pg/mL). The average intra-assay and inter-assay coefficients of variation for plasma NE and Epi was less than 5%.

Plasma NRG was measured using DuoSet ELISA development system (R&D cat# DY377) as previously described [11]. Briefly, a 96-well plate (Costar #9017) was coated with the capture antibody overnight at room temperature on a plate shaker. Capture antibody was washed, and the plate was blocked with blocking buffer as described in the package insert. A standard curve was generated using the lyophilized NRG according to manufacturer’s instructions with 2% normal goat serum (Santa Cruz #sc-2028). The standards and diluted samples (1:3 in 1% BSA/PBS) were added to each well and incubated for two hours. After serial washing, the detection antibody was added, incubated at room temperature and then washed. Streptavidin-HRP was added and incubated on the plate shaker for 30 minutes at room temperature. The plate was washed and then the substrate solution (Thermo Scientific 1-Step Ultra TMB-ELISA cat # 34028) was added for ten minutes, protected from light. Sulfuric acid stop solution was added, and the absorbance was read at 450 nm using a spectrophotometric plate reader.

Aliquoted samples were run in duplicates and the average of the two values was used in the analysis. Plasma NRG detection limit ranged from 0.3 to 30 ng/ml. None of the samples were below the detection limit. The average intra-assay coefficient of variation for plasma NRG was <10%, and the inter-assay coefficient of variation was 5.1%.

### Cardiac function

Cardiac function was assessed by two-dimensional echocardiography during study visits, pre-chemotherapy and after three months of TZB/LAP treatment. Quantitative biplane Simpson’s method was used to monitor changes in LVEF and to minimise intra-observer variability. Additional longitudinal cardiac function data were collected from the electronic medical record from echocardiograms that were performed for routine surveillance for chemotherapy cardiotoxicity. ‘Cardiac Function Decline’ was defined as the absolute maximal drop in LVEF during the follow-up period of up to three years. ‘Cardiac dysfunction’ was defined as an absolute reduction of 10% in LVEF from baseline, or a drop below 50% (lower limits of normal for the accredited Intersocietal Commission for the Accreditation of Echocardiography Laboratories Core Lab).

### Statistical analysis

Continuous variables were summarised as mean ± SD. Paired *t*-tests were used to test for differences in pair before vs. after data. Categorical variables were summarised as count and percentages. A *p*-value <0.05 was considered statistically significant. Statistical analysis was performed with statistical software SPSS Statistics version 18.0 (SPSS Inc, Chicago, Illinois, USA). Graphs were created with GraphPad Prism 5.02 (GraphPad Software, San Diego, California, USA).

## Results

### Characteristics of study patients

The 15 study patients were all Caucasian women (48 ± 9 years), with one subject (6%) having Hispanic ethnicity. The majority had early stage BC (66% Stage I), 38% had a history of hypertension, 7% had diabetes mellitus, 7% had a family history of HF, 7% had a history of coronary artery disease, and 27% had used tobacco (Table 1).

The most common chemotherapy regimen was a combination regimen of taxol, carboplatin, and TZB (TCH). A small percentage of women were prescribed beta-blockers (20%) or ACE-inhibitors (13%) prior to their treatment (Table 1). There was no measure of outpatient medication adherence in the study.

### Hemodynamic changes

There was a statistically significant increase in blood pressure but no significant change in heart rate after three months of TZB/LAP treatment (Table 2).

### Plasma catecholamines and neuregulin

**Figure 1.**
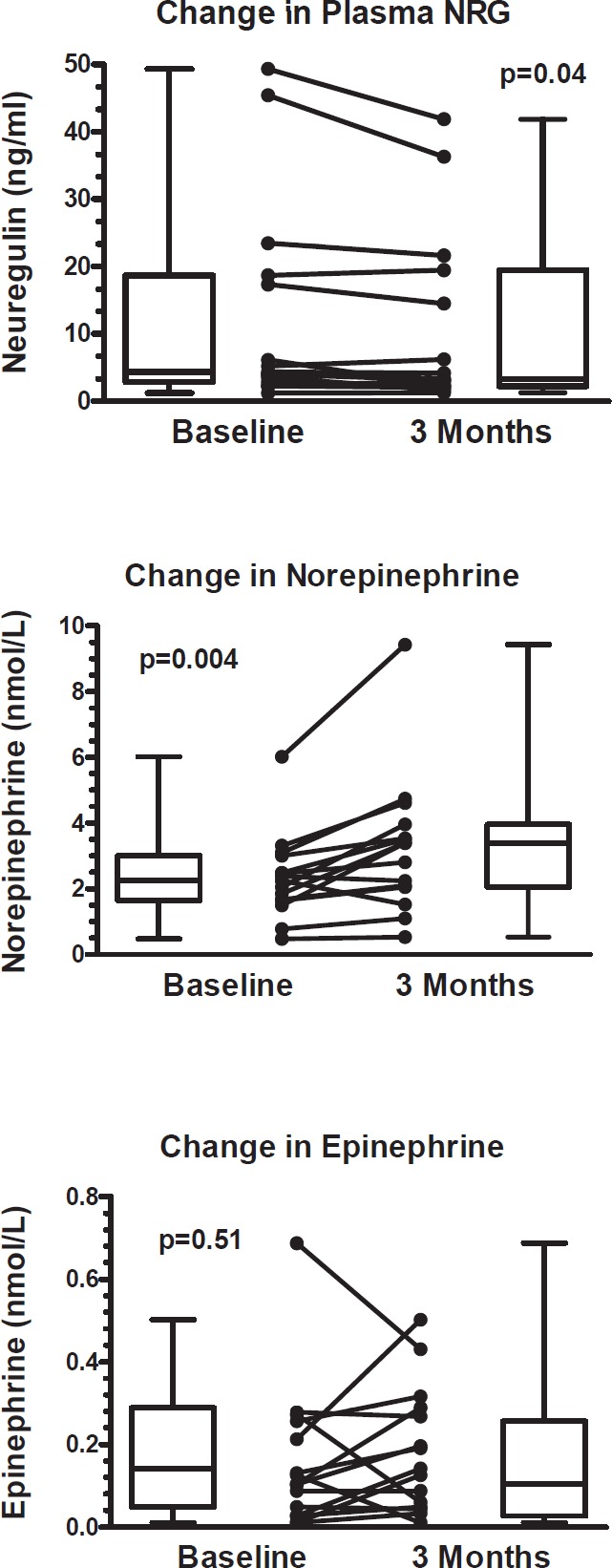
Change in circulating neuregulin, norepinephrine, and epinephrine from anti-HER2+ treatment. Upper and lower limit of box represent the 25th and 75th percentile and the whiskers represent the 95% confidence interval. *p* values were calculated with paired *t*-tests.

Compared to baseline, three months of anti-HER2 treatment significantly increased plasma NE 2.334 ± 1.294 nmol/L vs. 3.262 ± 2.103 nmol/L; *p* = 0.004, (395 ± 219 pg/ml vs. 551 ± 355 pg/ml; *p* = 0.004), significantly decreased plasma NRG (12.7±15.7 ng/ml vs. 10.9 ± 13.3 ng/ml; *p* = 0.04), but did not change plasma EPI 0.183 ± 0.151 nmol/L vs. 0.159 ± 0.174 nmol/L; *p* = 0.519, (34 ± 28 pg/ml vs. 29 ± 32 pg/ml; *p* = 0.51) (Table 2 and Figure 1). Since EPI levels did not change, this confirms that the change in NE levels reflects intrinsic sympathoneural tone and not spurious release from the adrenal gland.

### Cardiac function

**Figure 2.**
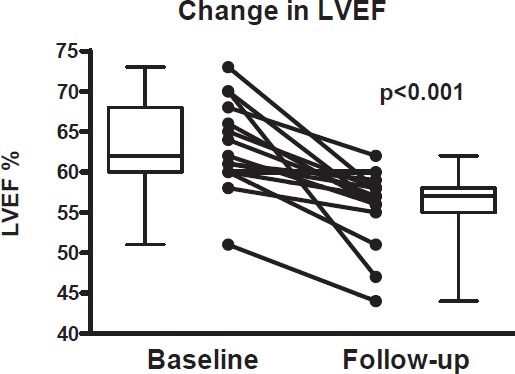
Left ventricular ejection fraction (LVEF) at baseline, and the lowest LVEF in three-years of follow-up with anti-HER2+. The upper and lower limit of box represent the 25th and 75th percentile, and the whiskers represent the 95% confidence interval. The *p* value was calculated with a paired *t*-test.

The cardiac function decline for the entire cohort was −7.9 ± 6.6%. ‘Cardiac dysfunction’ was observed in three (20%) patients (Figure 2). None of these three patients who developed ‘cardiac dysfunction’ were on a beta-blockers or ACE-inhibitors.

## Discussion

Since the US Food and Drug Administration approved the first monoclonal antibody for the treatment of HER2+ BC (TZB) in 1998, other anti-HER2 agents have emerged on the market. Anti-HER2 agents have significantly improved the mortality outcomes for HER2+ BC patients. Due to the success of anti-HER2 agents, epidemiologic studies demonstrate an eight-fold increase in the use of TZB from 2000 to 2007 with an expansion of its use to older BC patients with more cardiovascular risk factors than were studied in the pivotal clinical trials [6, 12]. These studies found an increased incidence of HF and subclinical cardiomyopathy with anti-HER2 agents, and raised the concern that the degree of cardiotoxicity is under-recognised.

Due to the rising rates of HF and subclinical cardiomyopathy with TZB, strategies for understanding, monitoring, treating, and, ideally, preventing anti-HER2 associated cardiotoxicity are urgently needed [13]. The mechanisms of cardiotoxicity are different between anthracycline-based chemotherapies versus targeted anti-HER2 agents such as TZB. One of the main causes of cardiotoxicity with anthracyclines results from increased free-radical formation and oxidative stress that causes permanent myocyte apoptosis and necrosis. In contrast, it is hypothesised that TZB causes cardiotoxicity by blocking the HER2/ERBB2 receptor-ligand signalling within cardiac myocytes disrupting critical intracellular signalling required for normal myocyte homeostasis, function, and repair. The recent finding that NRG/HER signalling plays a role in central and peripheral control of vasomotor tone and sympathetic output provides an alternative mechanism for adverse cardiovascular effects of HER2-targeted therapies. Indeed, we found that circulating NRG levels drop during anti-HER2 treatment with a corresponding increase in sympathetic tone (increase in blood pressure and NE) and a significant drop in LVEF over time. Therefore, HER2-targeted therapy may cause cardiac dysfunction both through the disruption of NRG/HER signalling, which is required not only for myocyte growth and repair but also cause a chronic increase in afterload, which can lead to a decline in ejection fraction. To our knowledge, this is the first evidence that NRG/HER signalling regulates vasomotor tone in humans.

The current strategies to monitor chemotherapy-associated cardiac dysfunction have relied on circulating levels of cardiac-specific troponin (cTnI) [14]. While TZB-like antibodies do not induce cell death directly [15], the combined increase in NE with decline in NRG may be sufficient to cause myocyte loss [16] explaining the observation that changes in high sensitivity cTnI can be detected during TZB therapy [17]. While this study was too small to determine whether NRG, NE or blood pressure predicts subsequent cardiac dysfunction, it supports the novel concept that TZB-associated cardiac dysfunction is in part due to disruption of the physiologic role of NRG/HER2 receptor signalling in control of vascular function.

Previous investigations have indicated the utility of beta-blockers in the treatment of chemotherapy-associated cardiac dysfunction [18, 19]. There are some data with regards to beta-blocker therapy improving cardiac function in the setting of TZB, as well as allowing a patient to be re-challenged with the same chemotherapy while maintaining stable cardiac function [20]. Currently, there is an ongoing prospective randomised clinical trial (MANTICORE 101 Breast, NCT01016886) evaluating the utility of prophylactic beta-blockers or ACE-inhibitors in preventing TZB associated cardiac dysfunction [21]. Since TZB appears to cause an increase in sympathoneural tone (with increased plasma NE), it is possible that beta-blockers may prevent TZB-associated cardiotoxicity by blunting the effects of increased sympathoneural tone [22]. Larger studies are needed to confirm the findings from this small study, and to more fully explore the role of sympathoexcitation in TZB-induced cardiac dysfunction.

## Conclusions

The current study demonstrates that Anti-HER2 treatment causes an increase in NE, blood pressure with a corresponding decrease in NRG; suggesting that the inhibition of NRG-HER2 signalling leads to increased sympathoneural tone. This is the first study in humans that suggests an increase in sympathetic tone via the NRG-HER2 pathways may be a key mechanism causing cardiac dysfunction in patients treated with TZB or LAP. Larger studies are needed to confirm these results and determine if cardiac dysfunction is attenuated with the use of beta-blockers.

## Limitations

This was a small proof of concept study, and it was therefore not adequately powered to determine if circulating NRG or NE predict cardiotoxicty. The investigators were also constrained by resources and unable to measure plasma catecholamines at multiple time points during treatment or able to have age-matched healthy controls. Additionally, our cohort was entirely Caucasian, limiting the generalisability. The study was performed at a tertiary-care, urban academic centre in Tennessee, and therefore is subject to a referral bias. These findings need to be reproduced in a larger cohort, with age-matched healthy controls, and in different racial and ethnic groups.

## Figures and Tables

**Table 1.** Patient characteristics of HER2+ breast cancer patients (*n* = 15).

**Average Age (years)**	48 ± 9 (range: 31–60)
**Average BMI**	29 ± 6 (range: 21–37)
**Stage of Breast Cancer (%)**	
I	66
II	17
III	17
**Cardiac Risk Factors (%)**	
Hypertension	38
Coronary Artery Disease	7
Diabetes	7
Positive Tobacco History	27
Family History of Heart Failure	7
**Chemotherapy**	
AC+TZB (%)	13
TCH (%)	53
AC+LAP	13
Other combination TZB+LAP (%)	20
**Medications**	
Beta-blocker (%)	20
ACE-inhibitor (%)	13

AC: Anthracycline; TZB: Trastuzumab; LAP: Lapatanib;

T: taxol; TCH: taxol, carboplatin, and trastuzumab combination

**Table 2.** Change in heart rate, blood pressure, catecholamine, and neuregulin from treatment of HER2+ breast cancer (n = 15).

Mean ± SD	n	Pre HER2+ Rx:	Post HER2+Rx:	*p* values
LVEF %	15	63 ± 6	56 ± 5^*^	<0.001
HR (BPM)	15	73 ± 12	77 ± 10	0.146
SBP (mm Hg)	15	110 ± 10	120 ± 16	0.049
DBP (mm Hg)	15	67 ± 14	77 ± 10	0.009
Norepinephrine (nmol/L)	15	2.334 ± 1.294	3.262 ± 2.103	0.004
Epinephrine (nmol/L)	15	0.183 ± 0.151	0.159 ± 0.174	0.519
Neuregulin (ng/ml)	15	12.7 ± 15.7	10.9 ± 13.3	0.036

Data are presented as mean + SD. *p* values reported for paired *t*-tests.

Maximal change in LVEF during follow-up
